# Mesmerism

**Published:** 1845-01

**Authors:** 


### MESMERISM.

We do not know whether to congratulate or condole with, the talented Heroine of Political Economy on the strange dream that has come over her soul. It appears that Miss Martineau recovered her health and—we were nearly saying—lost her senses! But this is not the case—she has acquired an additional sense—CLAIRVOYANCE! Her maid, BETTY, placed her hand on her mistress's ivory forehead, and, presto, a STEAM-TUG that was passing, became metamorphosed into a ship of celestial glory, fringed with gold and silver, and fit to be "a God-head's dwelling."

It's all in my eye BETTY MARTIN—EAU!

Betty, however, is no fool. She prescribed ale and brandy and water to her mistress, instead of opium-eating, and the change resulted in the best effects. Harriet's Mesmeric dreams will prove a god-send to the animal magnetisers, and will command more attention among the old women of both sexes than her Political Economy and her "Preventive Checks." But it won't do!

It will be the wonder of the day—perhaps of nine days—and then sink into oblivion with the exploits of Miss Okey.

---

### BIBLIOGRAPHICAL RECORD.

